# De Novo Computational Design of a Lipase with Hydrolysis Activity towards Middle-Chained Fatty Acid Esters

**DOI:** 10.3390/ijms24108581

**Published:** 2023-05-11

**Authors:** Jinsha Huang, Xiaoman Xie, Zhen Zheng, Luona Ye, Pengbo Wang, Li Xu, Ying Wu, Jinyong Yan, Min Yang, Yunjun Yan

**Affiliations:** Key Laboratory of Molecular Biophysics, Ministry of Education, College of Life Science and Technology, Huazhong University of Science and Technology, Wuhan 430074, China; huangjinsha@hust.edu.cn (J.H.); xiexm88@163.com (X.X.); 15522311733@163.com (Z.Z.); d201980534@hust.edu.cn (L.Y.); wangpb@hust.edu.cn (P.W.); wuying2010@hust.edu.cn (Y.W.); yjiny@126.com (J.Y.); ymyangmin@hust.edu.cn (M.Y.)

**Keywords:** lipase, de novo design, *theozyme*, laboratory-directed evolution, hydrolysis activity, middle-chained fatty acid esters

## Abstract

Innovations in biocatalysts provide great prospects for intolerant environments or novel reactions. Due to the limited catalytic capacity and the long-term and labor-intensive characteristics of mining enzymes with the desired functions, de novo enzyme design was developed to obtain industrial application candidates in a rapid and convenient way. Here, based on the catalytic mechanisms and the known structures of proteins, we proposed a computational protein design strategy combining de novo enzyme design and laboratory-directed evolution. Starting with the *theozyme* constructed using a quantum-mechanical approach, the theoretical enzyme-skeleton combinations were assembled and optimized via the Rosetta “inside-out” protocol. A small number of designed sequences were experimentally screened using SDS-PAGE, mass spectrometry and a qualitative activity assay in which the designed enzyme 1a8uD_1_ exhibited a measurable hydrolysis activity of 24.25 ± 0.57 U/g towards p-nitrophenyl octanoate. To improve the activity of the designed enzyme, molecular dynamics simulations and the RosettaDesign application were utilized to further optimize the substrate binding mode and amino acid sequence, thus keeping the residues of *theozyme* intact. The redesigned lipase 1a8uD_1_–M8 displayed enhanced hydrolysis activity towards p-nitrophenyl octanoate—3.34 times higher than that of 1a8uD_1_. Meanwhile, the natural skeleton protein (PDB entry 1a8u) did not display any hydrolysis activity, confirming that the hydrolysis abilities of the designed 1a8uD_1_ and the redesigned 1a8uD_1_–M8 were devised from scratch. More importantly, the designed 1a8uD_1_–M8 was also able to hydrolyze the natural middle-chained substrate (glycerol trioctanoate), for which the activity was 27.67 ± 0.69 U/g. This study indicates that the strategy employed here has great potential to generate novel enzymes exhibiting the desired reactions.

## 1. Introduction

Lipase is a widely used multifunctional enzyme with great potential in industries and applications such as the fat and petroleum processing industry, the oleochemical industry, the food industry, detergents, the pulp and paper industry, environmental management, tea processing, biosensors, and cosmetics and perfumes [1,2,3]. Despite these advantages and growing industrial interest, wild-type (wt) lipases are generally unsuitable for harsh environments or emerging reactions [4,5]. The most efficacious way to overcome these challenges is to search for novel enzymes with specific functions from the natural environment or to modify existing enzymes to obtain the desired functions [6,7,8,9]. However, with respect to labor consumption, it is much more efficient to modify the natural proteins at the molecular level than to mine new enzymes from nature [10,11].

As reported, designing enzymes with new or wild-like catalytic properties is a long-standing and elusive goal [12]. Starting with Breslow’s pioneering research in 1995 [13], research on artificial enzymes has had a long history characterized by remarkable progress in computational and experimental methodologies [14,15,16,17]. De novo enzyme design protocol is proposed to explore the extensive sequence space beyond the evolutionary pathway [18,19] because wild-type enzymes occupy only a fraction of the protein sequence space [20]. Baker’s group [21] developed an “inside-out” strategy to construct artificial enzymes using the Rosetta program: *theozyme*-containing catalytic motifs were first established and embedded into a robust protein backbone, followed by the optimization of the amino acids in the active pocket not participating directly in catalysis [22,23]. With the above-mentioned strategy, Baker et al. successfully created a series of enzymes (Retro-aldolase, Kemp eliminase and Diels-Alderase) from scratch, exhibiting orders of magnitude higher measured rates (catalytic rate constant/uncatalyzed rate constant, *k*_cat_/*k*_uncat_) against natural ones [24,25,26]. Furthermore, Fleishman et al. [27] proposed a general approach to design a highly active enzyme based on an automated combinatorial backbone assembly and sequence design, which has been applied to two unrelated families of enzymes with TIM-barrel folds (glycoside hydrolase 10 (GH10), xylanases and phosphotriesterase-like lactases (PLL)). Therein, using a set of homologous but structurally distinct enzyme structures, four designs with similar activity (*k*_cat_/*K*_M_, M^−1^·s^−1^) as the natural enzymes were obtained [27]. Recently, Ożga and Berlicki et al. [28] successfully constructed an artificial retro-aldolase using a miniprotein of truncated sequence with 43 amino acids retrieved from the C-terminus of the MvaT protein, and the reaction rate *k*_cat_/*k*_uncat_ of the designed peptide 20 was 5500. The approaches mentioned have offered new perspectives on the construction of artificial esterase/lipase.

Recently, numerous studies have been conducted to make progress in esterase/lipase design. Richter et al. [22] have explored the minimal structure for a highly proficient esterase, including Cys-His dyads and a backbone hydrogen bond to stabilize the oxyanion; however, the dyads were not correctly formed in the designed enzymes. Rajagopalan et al. [23] later designed nascent catalysts with organophosphate reactivity which simultaneously parsed out the contribution of the Ser in the nucleophilic catalysis. In our lab, Li et al. [29] also made efforts in the field. Our designed esterases displayed significant lytic activities towards short-chain length p-nitrophenyl (pNP) acetate, which encouraged us to extend the approach to construct artificial lipases targeting medium–long-chain substrates.

However, in the process of de novo enzyme design, the catalytical residues were placed in natural scaffolds that did not exhibit the desired activity, leading to decreased catalytic efficiency at several orders of magnitude as that of the natural enzyme [26,27,29]. Therefore, repeated laboratory evolution and structure-based recombination have been exploited to improve the stability and/or catalytic activity of artificial enzymes with modularity [30,31,32,33]. To give an example of this, Barber-Zucker et al. [34] used FuncLib calculations to design high-redox potential laccases for high functional diversity, suggesting that it is possible to reshape the biocatalytic functions.

In this study, we proposed a general design frame for artificial lipase based on hydrolytic enzyme backbones towards the medium-chain length substrate using the RosettaEnzymeDesign protocol paired with subsequent laboratory evolution. De novo designed artificial enzymes were successfully obtained.

## 2. Results

### 2.1. Computational Design of Enzymes from Scratch

**Figure 1:**
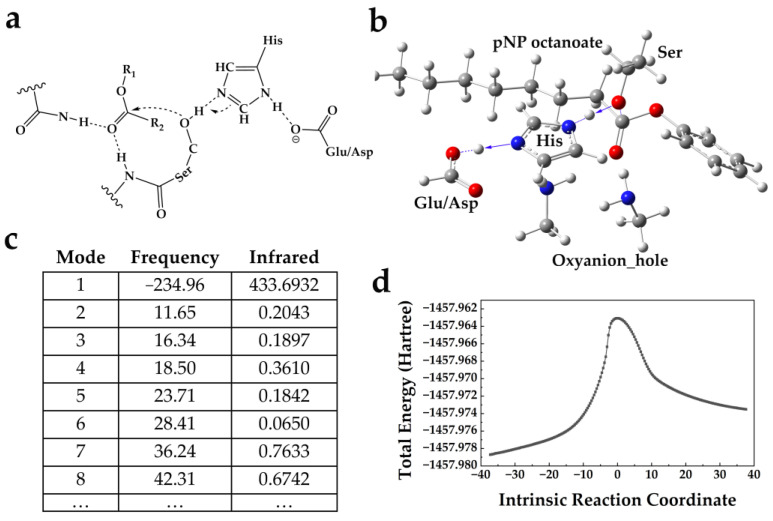
Construction of *theozyme*. (**a**) Illustration of *theozyme* geometry. The geometric parameters of precatalytic gating motion were derived from the previously reported data in Ser hydrolase [22,23,29,35]. (**b**) Quantum mechanically optimized catalytic motifs used in design. The residues and pNP octanoate are shown in ball-and-stick. Atoms of carbon, oxygen, nitrogen and hydrogen are shown in gray, red, blue and white, respectively. (**c**) Vibrational frequency calculations of transition state. (**d**) Total energy of the system calculated along intrinsic reaction coordinate. The energy of transition state exhibited the highest along the reaction pathway.

According to previous studies [22,23,29,35], the transition state with ideal active sites could be generated from quantum mechanics/molecular mechanics (QM/MM) calculations. Here, the key transition state of acylation, in which the carbonyl carbon of the ester substrate is nucleophilic attacked by the Ser, was the focus in constructing a predefined arrangement of catalytic functional residues in a stripped-down version (*theozyme*) [23,36]. As shown in Figure 1a, it consists of a Ser–His–Asp/Glu triad paired with an oxyanion hole formed by two NH groups on the neighboring backbone.

Additionally, given the high computing resource costs and requirements in QM/MM calculations, the entire residues of *theozyme* were simplified and replaced with the catalytic side chains, as depicted in Figure 1b. Meanwhile, the results of the vibrational frequency (Figure 1c) and intrinsic reaction coordinate (Figure 1d) illustrated that the conformation of the transition state along the pathway of the Ser nucleophilic attack was properly constructed.

**Figure 2:**
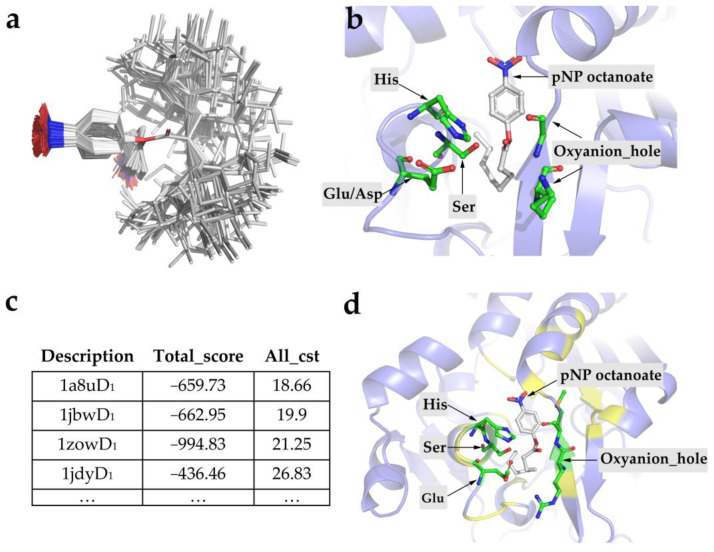
(**a**) A theoretical conformer ensemble of pNP octanoate superimposed over the carbonyl carbon and shown in sticks. Atoms of carbon, oxygen, nitrogen and hydrogen are shown in gray, red, blue and white, respectively. (**b**) An example of design model outputted by RosettaMatch. The active sites are colored in green and shown in ball-and-stick. Moreover, the rest of the residues in their equivalent scaffold are colored in slate and shown as a cartoon. (**c**) Total scores and the penalty scores (all_cst) of the top-ranked designs calculated using RosettaDesign. (**d**) An example of a crystal structure of the active designs outputted by RosettaDesign. The catalytic sites of the structure are shown in green, residues introduced into RosettaDesign are shown in yellow, and the remaining residues are colored in slate. The substrate is marked in white.

A theoretical conformer ensemble of pNP octanoate superimposed over the carbonyl carbon was shown in Figure 2a. The substrate rotamers together with the structure of *theozyme* were converted to a constraint file (Doc1 in the Supplemental Material), which can be recognized by the Rosetta program. Then, RosettaMatch was employed to output the grafted models by searching for protein backbones to accommodate the *theozyme* in a set of 1208 protein scaffolds from EzCatDB database, as listed in Table S1. The initial calculations indicated that the positions providing a more stable environment for the catalytic residues could be found for the *theozyme* rearrangement (Figure 2b). Afterwards, the RosettaDesign application outputted the refined models (Figure 2c,d). During the process, the Rosetta energy function and conformational sampling of the side-chain rotational isomers with flexible backbones were used to redesign the binding sites of the pNP octanoate. By changing the residues around the above-matched ligands, the functional interaction between the catalytic residues and the substrate was optimized and the affinity for the ligand was improved [21]. Meanwhile, the top 10 ranked designs were used as the input file for the next round of sequence design, and the process was reiterated until the sequence was almost unchanged in five consecutive designs [29]. Consequently, based on the final penalty scores for the constraints (Figure 2c) and visual inspection of the designed structures (Figure 2d), a small number of top-ranked sequences (1a8u-based lipase, 1a8uD_1_; 1jbw-based lipase, 1jbwD_1_; 1jdy-based lipase, 1jdyD_1_; 1zow-based lipase, 1zowD_1_) were chosen for experimental verification.

### 2.2. Preliminary Activity Screening

**Figure 3:**
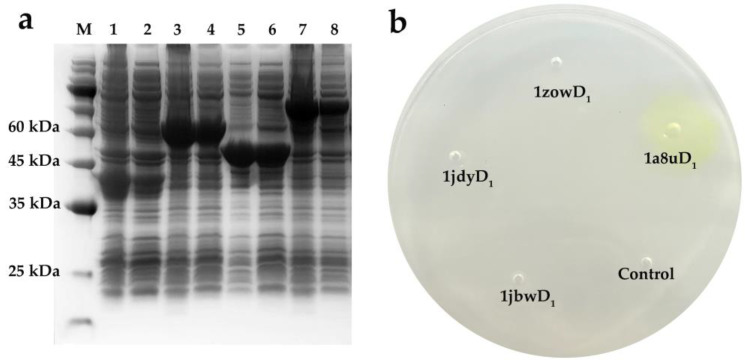
Experimental data of de novo designed enzymes. (**a**) SDS-PAGE of de novo designed enzymes using 15% precast mini polyacrylamide gel. Lane M: standard protein molecular weight (180/140/100/75/60/45/35/25/15/10 kDa); lane 1: supernatant of 1a8uD_1_; lane 2: inclusion bodies of 1a8uD_1_; lane 3: supernatant of 1jbwD_1_; lane 4: inclusion bodies of 1jbwD_1_; lane 5: supernatant of 1zowD_1_; lane 6: inclusion bodies of 1zowD_1_; lane 7: supernatant of 1jdyD_1_; lane 8: inclusion bodies of 1jdyD_1_. (**b**) Qualitative analysis of designed enzymes based on colorimetric activity assay. The lysates of enzymes were separately added into the corresponding hole in an agarose Petri dish. The area with active designs turned yellow, while the area containing the blank solution and inactive proteins displayed no color change.

Genes encoding for the four designed enzymes were codon-optimized using the codons preferred for high-level expression in *E. coli* and cloned into a pET-28a (+) vector for expression in *E. coli* BL21 (DE3). As shown in Figure 3a, the target proteins were effectively detected in the cell lysate of the four designed enzymes. Although inclusion bodies were formed, the selected designs (1a8uD_1_, 1jbwD_1_, 1jdyD_1_, 1zowD_1_) could be efficiently soluble expressed in *E. coli*. In addition, the peptide sequences of in silico designs excised from the blue-stained gel were identified by Nano LC-MS/MS to further verify the correct expression. As described in Tables S2 and S3, the total number of unique peptides recovered from the designed enzymes (1a8uD_1_, 1jbwD_1_, 1jdyD_1_, 1zowD_1_) were 14, 22, 45 and 14, which respectively accounted for 44.44% (124 out of 279 amino acids), 65.02% (277 out of 426 amino acids), 76.51% (430 out of 562 amino acids) and 72.76% (227 out of 312 amino acids) of the sequence. The activity was qualitatively assessed by observing the chromogenic phenomenon during the hydrolysis reaction of the pNP ester. As shown in Figure 3b, the pore with 1a8uD_1_ and its surrounding area turned yellow, showing a measurable hydrolysis capacity towards the pNP octanoate. However, there was no coloration in or around the holes with the addition of control samples or the designed enzymes (1jbwD_1_, 1zowD_1_ and 1jdyD_1_), indicating no hydrolytic activity.

### 2.3. Computational Redesign of 1a8uD_1_ Based on Its Backbone

**Figure 4:**
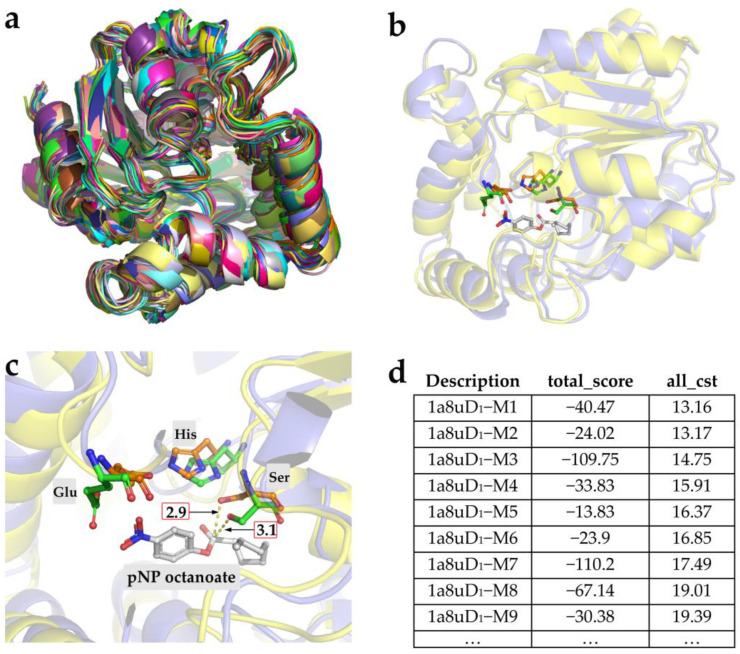
Computational data of redesigned enzymes. (**a**) Skeleton set of 1a8uD_1_ generated using MD and superimposed over the initial structure. (**b**) Structural comparison of the redesigned model (1a8uD_1_–M8) with that of the 1a8uD_1_. (**c**) Local visualization of the catalytic triad of the redesigned model (1a8uD_1_–M8) with that of the 1a8uD_1_. The numbers in the red box represents the nucleophilic attack distance. (**d**) The total score and penalty scores (all_cst) of the redesigned 1a8uD_1_ calculated using RosettaDesign. The redesigned models were predicted based on the RosettaEnzymeDesign protocol by restricting certain residues of *theozyme*. The 1a8uD_1_ is colored in slate, and the redesigned model (1a8uD_1_–M8) is colored in yellow. The catalytic residues and substrate are represented in ball-and-stick. The catalytic residues of 1a8uD_1_ and 1a8uD_1_–M8 are marked in green and orange, respectively. The substrate is marked in white.

Although new active sites capable of catalyzing the cleavage of the substrate have been artificially introduced into the selected backbone with the computational design, the enzymatic activity yielded was somewhat unsatisfactory [27,29]. To further improve the catalytic capacity, we attempted to redesign the amino acids around the active site of the designed enzyme using both MD simulations and the RosettaDesign application [37,38,39,40]. MD simulations were first applied to generate a skeleton library of 1a8uD_1_ as a starting point for enzyme design (Figure 4a). The constraints to keep the substrate in a reactive conformation were predefined based on the aforementioned *theozyme* using the QM/MM calculations (Figure 1b). The binding pocket was redesigned to accommodate the alternative residues using RosettaDesign application. Meanwhile, Rosetta energy functions and Monte Carlo algorithms were applied to filter for designs with reduced overall energy change and compatible mutation sets (Figure 4b,c). From the local visualization of the catalytic triad of the redesigned model (1a8uD_1_–M8) and 1a8uD_1_ in Figure 4c, it is apparent that the distance (nucleophilic attack distance) between the carbonyl carbon of the substrate and the hydroxyl oxygen of the Ser was altered after further optimization, which could significantly influence the catalytic potency. Finally, nine redesigned models (1a8uD_1_–M1 to 1a8uD_1_–M9) were selected for an experimental characterization based on the penalty scores of constraints (all_cst < 20) and a visual inspection of the structures (Figure 4d).

### 2.4. Activity Assay of the Redesigned Enzyme

**Figure 5:**
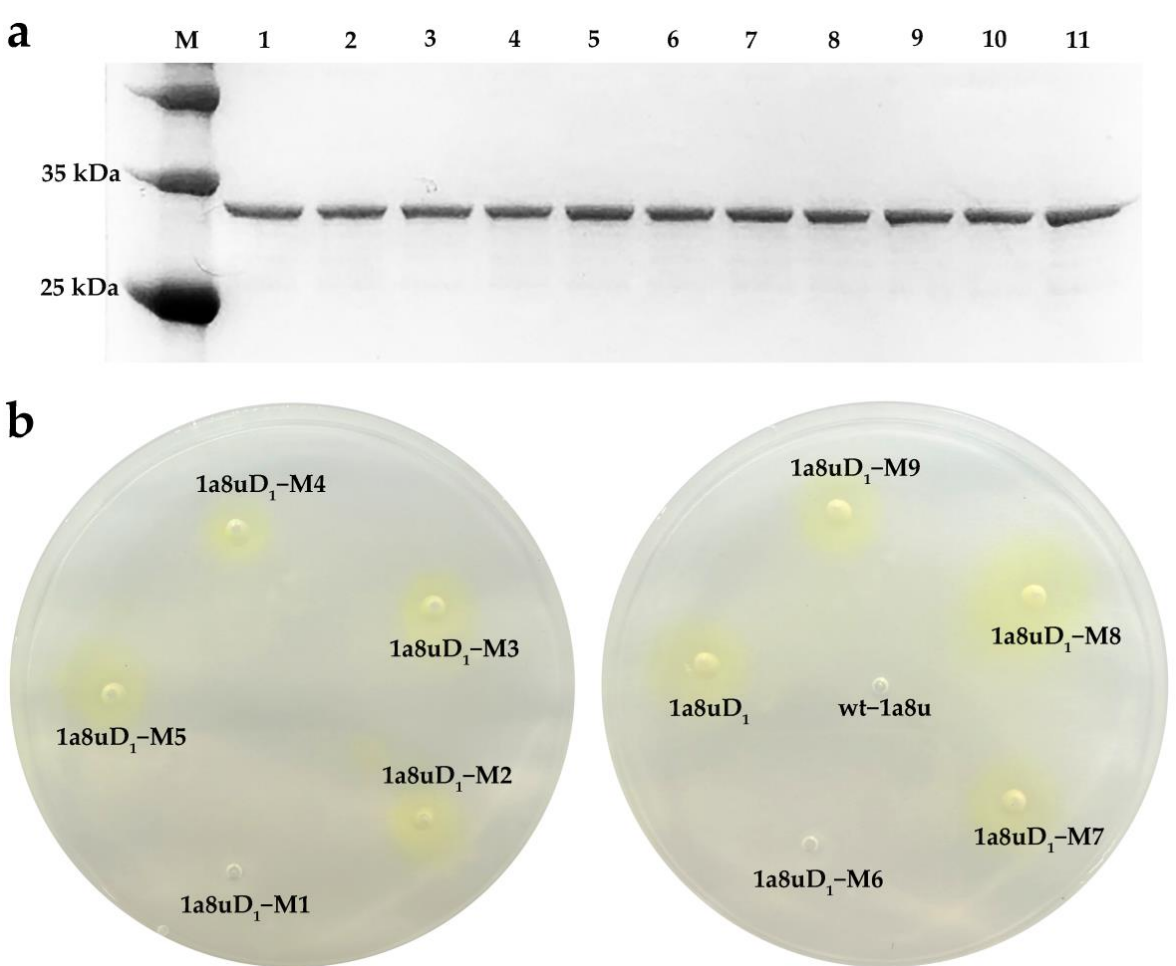
Experimental data of the redesigned enzymes. (**a**) SDS-PAGE of the redesigned 1a8uD_1_ using 15% precast mini polyacrylamide gel. Lane M: standard protein molecular weight; lane 1: wt–1a8u; lane 2: 1a8uD_1_; lane 3: 1a8uD_1_–M1; lane 4: 1a8uD_1_–M2; lane 5: 1a8uD_1_–M3; lane 6: 1a8uD_1_–M4; lane 7: 1a8uD_1_–M5; lane 8: 1a8uD_1_–M6; lane 9: 1a8uD_1_–M7; lane 10: 1a8uD_1_–M8; lane 11: 1a8uD_1_–M9. (**b**) Qualitative analysis of redesigned enzymes according to color change. The holes with active enzymes, and their surrounding area, turned yellow.

As illustrated in Figure 5a, the redesigned enzymes were soluble expressed in *E. coli* effectively and efficiently. Afterwards, the catalytic activity towards the pNP octanoate was detected by observing the chromogenic phenomenon during the hydrolysis reaction of the pNP ester. As seen in Figure 5b, there was no discoloration in or around the holes due to the addition of the redesigned 1a8uD_1_–M1/M6, indicating that the mutations caused the activity loss of the 1a8uD_1_. Further, the other redesigned enzymes exhibited measurable activity, as the holes and their surrounding area turned yellow. Furthermore, the activity of all the positive designs was also quantitatively measured according to the standard colorimetric method, and the results were depicted in Table 1. The specific activity of the 1a8uD_1_ was 24.25 ± 0.57 U/g, while certain redesigned catalysts exhibited improved activity after laboratory evolution; the mutant 1a8uD_1_–M8 performed the best enzyme activity of 81.07 ± 4.59 U/g, which was 3.34 times higher than that of the 1a8uD_1_. This phenomenon may be attributed to the nucleophilic attack distance being shortened from 3.1 Å to 2.9 Å (Figure 4c). Additionally, the template backbone protein wt–1a8u did not exhibit any activity to hydrolyze the pNP octanoate (Table S4), confirming that the observed hydrolytic activity of the designed lipases was derived from the designed motif of the *theozyme*.

To clearly unveil the source of the catalytic functionalities, the vital role of the components of the *theozyme* in the 1a8uD_1_–M8 was subjected to alanine mutagenesis, and all mutants were successfully expressed, as shown in Figure S1a. From the activity results in Figure S1b and Table S4, variants with a substitution of either residue in the *theozyme* exhibited completely abolished (mutation S99A and H31A) or greatly reduced (mutation E230A) the enzymatic activity. Thus, we conclude that the accuracy and activity of the preorganized *theozyme* is critical to the success of ab initio enzyme design because the removal of any of these functional amino acids would lead to a dramatic decrease in activity, a phenomenon also observed in other hydrolases [29,41,42].

### 2.5. Qualitative and Quantitative Evaluation of Positive Designed 1a8uD_1_ and 1a8uD_1_–M8 Using Glyceryl Trioctanoate as Natural Substrate

To explore the ability of the designed enzyme (1a8uD_1_ and 1a8uD_1_–M8) to hydrolyze the natural substrates, qualitative enzyme activity assays were performed using plates containing glyceryl trioctanoate, where an equal volume of Tris–HCl was used as a control. As shown in Figure S2, the mutant 1a8uD_1_ and 1a8uD_1_–M8 could form a clear transparent circle on the petri dishes containing glyceryl trioctanoate, and the diameter of the circle with the enzyme 1a8uD_1_–M8 was larger. Additionally, the activity towards the glyceryl trioctanoate was further quantified, as shown in Table 2. Similarly, the wt–1a8u was ineffective, while the designed enzymes (1a8uD_1_ and 1a8uD_1_–M8) all displayed some hydrolytic ability. The activity of the 1a8uD_1_ was 7.28 ± 0.45 U/g, and the activity of the 1a8uD_1_–M8 was 27.67 ± 0.69 U/g. Qualitative and quantitative analyses of the hydrolysis against the natural substrates indicated that the artificial lipase obtained using the combined framework of the RosettaEnzymeDesign could hydrolyze the natural ester and the resulting laboratory evolution method could further enhance the activity of the designed enzyme.

## 3. Discussion

Natural molecular evolution is highly efficiency to be able to react to this process by gradually accumulating random mutations. The process is slow, challenging and has an unsatisfactory success rate [43]. To glean the merits of natural selection in the laboratory, a computational-aided protein design strategy can be applied to shorten the evolutionary time frame (which would normally take millions of years) into a manageable experimental period [44]. Despite the benefits in the field of site-specific mutagenesis, enzyme design from scratch enables the direct introduction of multiple compatible point mutations into the catalytic core at a stroke, thereby overcoming the limitation in the number of potential substitutions and avoiding tremendous efforts in the laboratory [24,25,26,29].

Lipase (E.C.3.1.1.3) has been popular in directed evolution in recent decades due to its significance in biotech industries such as the food, pharmaceuticals, bioenergy and fine chemicals industries [45,46,47]. Li et al. [29] in our laboratory reported a de novo designed esterase with short-length chain ester (pNP acetate) hydrolysis activity according to the Rosetta “inside-out” design strategy. In this study, we proposed a more productive strategy to construct a new lipase with primitive hydrolysis activity towards the medium-length chain substrate (pNP octanoate) by combining the Rosetta enzyme design with laboratory evolution (MD simulations and RosettaDesign application). According to the qualitative detection of catalytic efficiency, the structure of the cofactor-free chloroperoxidases (PDB entry 1a8u) was handpicked as the active and stable skeleton. The optimal enzymatic activity of the artificial enzyme (1a8uD_1_–M8) obtained in this study was 81.07 ± 4.59 U/g, which is approximately one thousandth of that of the commercial lipase, like most ab initio designed enzymes [29]. Although the activity of the enzyme designed was far lower than that of commercial or natural enzymes, the method still paves the way for further evolution into promising and productive enzymes [48].

Since it is challenging to simultaneously optimize several processes (the binding of the substrate, the stabilization of the transition state and the release of the product) required in de novo enzyme design, many researchers have focused on one key property in ester catalysis of enzymes [23]. Given the calculation complexity and the available computational resources, we focused on the initiation process of nucleophile, in which the lone pairs of electrons generated by the side-chain groups attack the positively charged carbonyl carbon of the substrate, forming a covalent acyl intermediate [22,23,49]. As seen in Figure 1a,b, the Ser is selected as a better candidate for a nucleophile in our calculation. Although Ser or Cys is usually used as a nucleophile in natural lipases, the activation of the cysteine is much easier than that of the Ser (the pka values of Ser and Cys are 13 and 8, respectively) [22], leading to the hydrolysis of the enzyme intermediate and reducing the overall catalytic efficiency [12,23]. In addition, the Cys-His interaction is much weaker than that of the Ser–His and not sufficient to stabilize His in the desired conformation [23,26]. Therefore, to construct a more precise geometry of the *theozyme*, the typical Ser–His–Asp/Glu catalytic triad paired with an oxyanion hole formed by two NH groups on the neighboring backbone were used in our study (Figure 1).

Although the skeletons used in the study were not restricted to certain ones, their highly malleable and flexible channels offered a great potential to design new functions. Using the RosettaMatch and RosettaDesign calculations, the *theozyme* was placed in the appropriate locations in the skeleton, and the affinity towards the ligand was ensured by modifying the residues around the matching points (Figure 2b,d). The four top-ranked designs were synthesized and expressed in *E. coli*. Considering that the numerous mutations that occurred in the sequences of the scaffolds may compromise the folding and integrity of the backbone proteins, it is easier to form inclusion bodies and decrease the soluble expression level [21,24,29,50]. Furthermore, the four selected skeletons (1a8u, 1zow, 1djy, 1bjw) are thermophilic proteins with tight structures, which are stable enough to weaken the adverse effects of the mutation accumulation [51,52]. Hence, although there was a substantial inclusion body formation in the candidates, the soluble expression was at a detectable level (Figure 3a). Remarkably, only the variant 1a8uD_1_ displayed discernible hydrolase activity towards the pNP octanoate, and the others (1zowD_1_, 1djyD_1_, 1bjwD_1_) did not show any enzymatic activity (Figure 3b). However, the phenomena may be too complex to explain the evolutionary process through traditional evolutionary theory, due to the presence of up to 30 mutations in the binding pocket [44]. Nevertheless, the result represents the capacity to functionally design enzymes from scratch.

Notwithstanding, inspiring progress has been made in de novo enzyme design using the Rosetta3 protocol [53]. The computationally designed enzymes were much less active than the natural ones [21,48]. It was reported that conventional laboratory evolution has been successfully applied to evolve the initial enzyme into a more proficient catalyst simply through the iterative mutagenesis of certain sites rather than the introduction of a new active center [37,54,55]. As such, after optimizing the sequence space within the scaffold, the redesigned lipase (mutant 1a8uD–M8) displayed 3.34 times more hydrolytic activity towards the pNP octanoate than the original (Table 1). This result suggests that the substitutions only changed the conformation of the channel (in charge of binding the substrate for the catalysis), while the hydrophobic environment specific to the catalytic reaction was maintained unaltered. Additionally, because the substitutions inserted into the substrate channel through RosettaDesign calculations have complex epistatic relationships [44], the contribution of each individual mutation (other than those in the *theozyme*) was not analyzed using single-point mutations in this paper.

To date, the “inside-out” RosettaEnzymeDesign protocol was originally conceived for de novo design enzymes to catalyze new reactions. It has been successfully implemented in diverse scenarios in brief periods of time [27,44]. However, the result is usually unsatisfactory when only this design tool is used [29]. For instance, the catalytic activity of the 1a8uD_1_ designed in the top-down manner was only 24.25 ± 0.57 U/g. The subsequent laboratory evolution could help improve the unsatisfied catalytic activity of artificial enzymes to a certain extent [56]. In combination with MD simulations and the RosettaDesign application, an artificial design named 1a8uD_1_–M8 with significantly enhanced activity (81.07 ± 4.59 U/g) was obtained through further evolution. Moreover, the de novo designed lipase 1a8uD_1_ and 1a8uD_1_–M8 were also able to catalyze the hydrolysis reaction against the natural substrate (glycerol trioctanoate), in which the activity of the 1a8uD_1_–M8 was 3.80 times higher than that of the 1a8uD_1_. Therefore, the combinational method integrating the RosettaEnzymeDesign protocal and laboratory evolution may be suitable for the de novo design of artificial enzymes, which will assist in dealing with complex enzyme engineering problems quickly and efficiently.

## 4. Materials and Methods

### 4.1. General Information

All the reagents were commercially available and used as received: isopropyl β-D-1-thiogalactopyranoside (Solarbio, Beijing, China); kanamycin (Solarbio, Beijing, China); p-nitrophenyl octanoate (Sigma-Aldrich, St. Louis, MO, USA); plasmid mini kit I (Omega, Norcross, GA, USA); gel DNA extraction mini kit (Vazyme, Nanjing, China); Bradford protein assay kit (Tiangen, Beijing, China); 15% precast mini polyacrylamide gels (Genscript, Nanjing, China); and BeyoGold™ His-tag Purification Resin (Beyotime, Shanghai, China). All the other chemicals were purchased from China Pharmaceutical Chemical Reagent Co., Ltd. (Shanghai, China).

### 4.2. Computational Design of Enzymes from Scratch

(i) Construction of *theozymes*. pNP ester is the most commonly used model substrate in the study of ester hydrolysis as it can easily mimic the tetrahedral transition state and provide a carbonyl group (electrophile) to test the nucleophilicity of the Ser in the designed protein [22,23,49]. Here, the pNP octanoate was selected as the model substrate. In addition, quantum mechanics calculations have been reported to predict the transition state geometries along the ester hydrolysis reaction path [22,35,49]. In such calculations, the Ser usually acts as a nucleophile and is deprotonated by a hydrogen-bonded His, which acts as a general base [2,49]. The Asp/Glu, another hydrogen bond acceptor, is often employed to neutralize the charges on the His in the transition state and to orient the imidazole ring [22,23,35]. The highly conserved oxyanion hole is used to precisely identify the hydrogen bond donor and to stabilize the negative charge on the carbonyl oxygen of the tetrahedral intermediate, and almost all the natural lipases prefer to use the NH groups on the more rigid backbone rather than on the side chains [49,57,58]. Therefore, the *theozyme*, made up of a Ser–His–Asp/Glu triad plus one oxyanion hole composed of two residues, was computed using Gaussian 09 at the M06-2X/6-311++g(2d, 2p) level of theory [29,59]. The vibrational frequency and intrinsic reaction coordinates were calculated to evaluate the accuracy of the *theozyme*.

(ii) An ensemble of substrate rotamers. An ensemble of substrate (pNP octanoate) conformations was generated by employing an open-source cheminformatics toolkit—OpenBabel [60].

(iii) Protein skeleton library for de novo design. Based on existing computing power and our previous work [29,61], protein scaffolds with any Ser, His, Asp or Glu as catalytic residues in the EzCatDB database were selected as candidates for the transition state placement in the process of the de novo enzyme design.

(iv) Computational design based on the RosettaMatch and RosettaDesign applications. First, a constraint (cst) file, used as the input to Rosetta program, was generated by converting the coordinates of the *theozyme*. The parameter file containing the information about the bond angles, bond distances and dihedral angles, etc. was detailed in Doc S1 in Supplemental Material. After that, the RosettaMatch algorithm was used to accurately identify the attachment sites of the *theozyme* in the pre-selected skeleton set by simultaneously analyzing the transition state and the catalytically important groups [25]. The possible steric conflicts were then iteratively optimized using RosettaDesign by introducing mutations into the non-catalytic residues or rotating the amino acid sidechains surrounding the binding pocket in the scaffold–*theozyme* geometry [37]. Subsequently, RosettaDesign used a Monte Carlo algorithm to select mutations and structural changes, so that the various structural parameters of the designed enzymes were as close as possible to the nearly ideal geometry [22,38,39,40]. Finally, the outputs were sorted and screened on the basis of the following guidelines described in previous studies [29,37]: (1) the total energy should be negative, (2) the overall folding free energy changes of the designed enzyme and original scaffold were comparable, (3) the total score of the penalty energies for the constraints should be less than 30 REU (all_cst < 30), (4) large cavities should not appear in the structures of the designed proteins after the introduction of the mutations around the binding pocket (visualization). One or more were selected as the putative models of the designed enzymes to be evaluated and used for subsequent experimental characterization.

The code used for the RosettaEnzymeDesign protocol was depicted in Doc S2 in Supplemental Material.

Computational redesigned methods of designed enzymes.

To further enhance the catalytic activity of the favorably designed enzyme, laboratory evolution based on the molecular dynamics (MD) simulation and RosettaDesign application was employed to optimize and reshape the active pocket [37,40]. As starting points for Rosetta, an ensemble of scaffolds was generated by enumeration through GROMACS-based MD simulations. The MD simulations were implemented under GROMACS (version 2021.1) using an Amber99sb-ILDN protein force field and TIP3P water model on Linux-based workstations. The system was electrostatic neutralized with counter ions. Energy minimization (10 ps) was performed prior to NVT and NPT equilibration (100 ps) using the steepest descent method. Then, the final production stage was performed at 310 K for 100 ns with periodic boundary conditions and a time-step of 2 fs. In addition, the substrate rotamers library was generated by OpenBabel as described above and the Rosetta Design method (RosettaMatch and RosettaDesign) was in accordance with that described in section “Computational design of enzymes from scratch”.

### 4.3. Experimental Methods

(i) Construction, expression and purification of the designed enzymes. The genes encoding de novo enzymes were synthesized by Genscript between EcoRI and NotI in plasmid pET-28a (+) with an N-terminal His_6_, and *Escherichia coli* BL21 (DE3) was used as a host for gene expression. The protein expression was conducted in a Luria-Bertani medium, as described previously [29,33]. The cells (1 L) were harvested by centrifugation (5000× *g*, 10 min) and lysed by ultrasonication in a 50 mL solution containing 50 mM Tris, 0.5 mM EDTA and 50 mM NaCl. The lysates were then clarified by centrifugation and filtration. Subsequently, the clarified lysates were purified with 10 mL of His-tag purification resin, in which various concentrations of imidazole solution (2 mM, 4 mM, 10 mM, and 30 mM imidazole) were used for gradient elution from the Ni Sepharose Fast Flow column. After that, the proteins were treated three times in the dialysate (50 mM potassium phosphate buffer) at 4 ℃.

(ii) Identification of the designed enzymes. The molecular weights of the selected proteins were determined on SDS-PAGE using 15% precast mini polyacrylamide gels and stained using the eStain^®^ LG Protein Staining System (Genscript, Nanjing, China). Mass spectrometry was performed on the purified samples separated from the SDS-PGAE gel for protein identification at ProtTech, Inc. (Suzhou, China) using Nano liquid chromatography tandem mass spectrometry (Nano LC-MS/MS) peptide-sequencing technology commercially. Protein concentrations were estimated using the Bradford protein assay kit in triplicate, with bovine serum albumin as the standard.

(iii) Enzyme activity assays. Positive designed enzymes were preliminarily screened based on the qualitative assay according to our previous study [29]. Petri dishes with circular holes with a diameter of 4–5 mm were prepared using 1.5–2% agarose, 200 µL (100 mM) of pNP octanoate and 100 mL of Tris–HCl buffer (50 mM, pH 8.0). Then, each designed enzyme was added into its corresponding hole and incubated at 37 ℃ to observe the color changes in and around the holes. Additionally, through standard colorimetric determination, described previously [33,62], the pNP octanoate dissolved in the acetonitrile was utilized to measure the hydrolysis activity of the designed enzyme. In brief, the reaction mixture was prepared with 10 µL of emulsified pNP octanoate, 40 µL of ethanol, and an appropriate amount of enzyme dissolved in Tris–HCl buffer (50 mM, pH 8.0). The assay was kept at 37 ℃ for 5 min. The amount of enzyme consumed in releasing 1 µmol of p-nitrophenol per minute was considered one unit (U). A control with the addition of the same volume of Tris–HCl was used in this experiment. All the assays were carried out in triplicate, and the buffers used were prepared according to the experimental temperature.

(iv) Qualitative and quantitative evaluation of the active designed lipases (1a8uD_1_ and 1a8uD_1_–M8) using glyceryl trioctanoate. Petri dishes with circular holes with a diameter of 4–5 mm were prepared using 1.5–2% agarose, 10 mL/L glyceryl trioctanoate and 100 mL of Tris–HCl buffer (50 mM, pH 8.0). Then, a certain volume of the enzyme was added into its corresponding hole at 2 h intervals and incubated at 37 ℃ for 24 h to qualitatively analyze the activity according to the transparency circle. Furthermore, the enzymatic activity against the glyceryl trioctanoate was quantitatively determined according to previous report [63]. A reaction mixture containing 100 μL of triglyceride and an appropriate amount of the designed enzyme in 3 mL of 50 mM Tris–HCl buffer (pH 8.0) was incubated at 37 °C and 200 rpm for 24 h. Afterwards, the reaction was terminated by adding 10 mL of absolute ethanol, and the released fatty acids were neutralized with 10 mM of NaOH while using phenolphthalein as an indicator. The amount of the enzyme consumed in generating 1 µM of fatty acid per minute was defined as one unit (U) of enzymatic activity.

## 5. Conclusions

In the study, an “inside-out” computational approach was used to create an artificial lipase towards middle-chained fatty acid esters from scratch, which integrated the RosettaEnzymeDesign protocol and the laboratory evolution method, including molecular dynamic simulation. The successful design of the artificial lipase 1a8uD_1_–M8 indicated that the strategy is sufficient to generate a novel enzyme with a similar ester hydrolysis as that of natural ones. Because of the limited computational resources, detailed information about each transition state along the pathway of the reaction was not taken into account in our work, resulting in a less comprehensive screening with insufficient candidates. Therefore, automatically designing stable and complex enzymes with catalytic efficiency comparable to that of natural enzymes is possibly a long-term and elusive goal. Nevertheless, with the development of computational algorithms and the establishment of large computer clusters, it is foreseen that the integration of in silico design and screening into ab initio enzyme design or directed evolution can effectively and efficiently increase the mining depth while reducing the time-consuming and costly laboratory work to create industrially relevant biocatalysts.

## Figures and Tables

**Table 1:** Enzymatic activity towards p-nitrophenyl octanoate and protein concentration of the wild and designed enzymes expressed in the work.

Description	Concentration of Purified Protein (mg/mL)	Catalytic Activity (U/g) ^a^
wt–1a8u	0.83 ± 0.03	N/A
1a8uD_1_	0.62 ± 0.03	24.25 ± 0.57
1a8uD_1_–M1	0.54 ± 0.02	N/A
1a8uD_1_–M2	0.65 ± 0.03	43.03 ± 1.00
1a8uD_1_–M3	0.60 ± 0.03	36.63 ± 2.63
1a8uD_1_–M4	0.59 ± 0.04	20.47 ± 0.77
1a8uD_1_–M5	0.50 ± 0.02	32.13 ± 1.82
1a8uD_1_–M6	0.55 ± 0.02	N/A
1a8uD_1_–M7	0.63 ± 0.02	25.31 ± 1.07
1a8uD_1_–M8	0.63 ± 0.02	81.07 ± 4.59
1a8uD_1_–M9	0.62 ± 0.03	22.38 ± 0.92

^a^ Mean ± standard deviation.

**Table 2:** Hydrolytic activity of the designed enzyme against glyceryl trioctanoate.

Mutations	wt–1a8u	1a8uD_1_	1a8uD_1_–M8
Catalytic activity (U/g) ^a^	N/A	7.28 ± 0.45	27.67 ± 0.69

^a^ Mean ± standard deviation.

## Data Availability

No new data were created or analyzed in this study. Data sharing is not applicable to this article.

## Supplementary Materials

The following supporting information can be downloaded at: https://www.mdpi.com/article/10.3390/ijms24108581/s1.
