# Supplementary material for: De Novo Computational Design of a Lipase with Hydrolysis Activity towards Middle-Chained Fatty Acid Esters
Source: Int J Mol Sci. 2023 May 11;24(10):8581. doi: 10.3390/ijms24108581 (PMC10218306; doi:10.3390/ijms24108581)
Supplement: Supplementary file 1 [file ijms-24-08581-s001.zip › ijms-2384519-supplementary.pdf]

Supplemental Materials

De novo computational design of a lipase with hydrolysis activity towards middle-chained fatty acid esters

Jinsha Huang, Xiaoman Xie, Zhen Zheng, Luona Ye, Pengbo Wang, Li Xu*, Ying Wu, Jinyong Yan, Min Yang, Yunjun Yan*

Key Laboratory of Molecular Biophysics of the Ministry of Education, College of Life Science and Technology, Huazhong University of Science and Technology, Wuhan, People's Republic of China

*Corresponding authors:

Li Xu, xuli@hust.edu.cn;

Yunjun Yan, yanyunjun@hust.edu.cn;

Table of Contents

Table S1 Protein Skeleton Library used for RosettaMatch.

Table S2 Summary of computational and experimental data of the designed enzymes. The peptide sequences identified by Nano LC-MS/MS were highlighted with an underline. The designed triad and oxyanion hole were in italics and bold.

Table S3 Summary of the peptide sequences identified by NanoLC-MS/MS.

Table S4 Enzymatic activity of designed enzyme 1a8uD_1−M8 after alanine mutation in residue of catalytic triad.

Figure S1 Analysis of purified 1a8uD_1−M8 mutants (S99A, H31A, E230A) after alanine mutation of either residue in catalytic triad. (a) SDS-PAGE of purified 1a8uD_1−M8 mutants using 15% precast mini polyacrylamide gel. Lane M: standard protein molecular weight (180/140/100/75/60/45/35/25/15/10 kDa), lane 1: mutant S99A, lane 2: mutant H31A, lane 3: mutant E230A. (b) Qualitative analysis of 1a8uD_1−M8 mutants according to color change in comparison with that of designed lipases (1a8uD_1, 1a8uD_1−M8) using p-nitrophenyl octanoate as the substrate.

Figure S2 Qualitative analysis of wt−1a8u, mutant 1a8uD_1 and mutant 1a8uD_1−M8 on a petri dish with emulsified glyceryl trioctanoate (10 mL/L) for 24 h.

Doc S1 Constraint file used for Rosetta Enzyme Design.

Doc S2 The script for Rosetta Enzyme Design.

Table S1 Protein Skeleton Library used for RosettaMatch.

**Protein Skeleton Library (1208)**
1a0j 1amn 1b8n 1btx 1c5t 1cus 1dwe 1ejm 1fgs 1gbd 1h1y 1hxe 1kfj 1mio 1op8 1ppe 1qoq 1slu 1tnk 1v1m 1xzh 2c8v 2qnx 2xmg 3il7 4tpi 1a0l 1an1 1b8o 1bty 1c5u 1cuu 1dwo 1ejn 1fi8 1gbe 1h1z 1hxf 1kfk 1mkw 1orf 1ppf 1qoz 1slv 1tnl 1v1r 1xzi 2ccr 2qny 2xqf 3il9 4uag 1a2c 1anb 1b93 1btz 1c5v 1cuv 1dwp 1ekb 1fiw 1gbf 1h22 1hy1 1kfq 1mkx 1owd 1ppg 1qq4 1slw 1tom 1va4 1xzj 2cga 2qnz 2xqg 3lpr 4vgc 1a2o 1anc 1ba8 1bu8 1c5w 1cuw 1dwq 1ela 1fiz 1gbh 1h23 1hzp 1ki0 1mmj 1owe 1pph 1qqu 1slx 1ton 1vdk 1xzk 2cha 2qo0 2xqi 3min 4yas 1a2q 1and 1bav 1bui 1c5x 1cux 1dx5 1elb 1fj2 1gbi 1h8d 1i5k 1klt 1mn1 1owh 1ptk 1qr3 1smf 1tpa 1vfn 1xzl 2cpo 2qo1 2xqj 3ncl 5cha 1a3b 1ane 1bb0 1bwp 1c5y 1cuy 1dx6 1elc 1fle 1gbj 1h8i 1i99 1kol 1mn2 1owi 1pyt 1qrw 1som 1tpg 1vgc 1xzm 2ctb 2qx1 2xqk 3nps 5cpa 1a3e 1ao5 1bbr 1bwq 1c5z 1cuz 1e0d 1eld 1fmg 1gbk 1h9l 1iau 1kq7 1mnp 1owj 1q0p 1qrx 1spb 1tpk 1vjs 1yas 2ctc 2sbt 2xwb 3o9m 5est 1a46 1aos 1bcr 1bwr 1c8v 1cw2 1e34 1ele 1fn6 1gbl 1h9o 1ic6 1krn 1msk 1owk 1q3x 1qrz 1spj 1tpm 1vkl 1ycp 2cut 2sec 2xwj 3p8f 5gch 1a4w 1apq 1bcs 1bxz 1c9d 1cx9 1e35 1elf 1fn8 1gbm 1hag 1ihs 1kwm 1mtn 1oxm 1qa0 1qti 1st2 1tpn 1vot 1yfe 2dfp 2sga 2y1k 3p8g 5gds 1a50 1aq7 1bcu 1bzx 1c9p 1cxp 1e36 1elg 1fni 1gbt 1hah 1iht 1kyn 1mts 1oyv 1qad 1qtr 1sua 1tpo 1vq2 1yfm 2ebd 2sgd 2yas 3pmg 5hpg 1a5a 1aql 1bda 1c1m 1ca0 1d2v 1e37 1ell 1fp4 1gcd 1hai 1ik4 1l0z 1mtu 1p01 1qb1 1qy6 1sub 1tpp 1vsb 1yja 2eft 2sge 3bn9 3pnp 5lpr 1a5b 1aqn 1be6 1c1n 1ca8 1d3d 1e38 1elm 1fp6 1gct 1hao 1ild 1l1g 1mtv 1p02 1qb6 1r8l 1suc 1tps 1vxo 1yjb 2est 2sgf 3btd 3prk 5ptp 1a5g 1ar1 1be8 1c1o 1cbw 1d3p 1e3q 1elt 1fpc 1gdn 1hap 1ilz 1l4d 1mtw 1p03 1qb9 1rfn 1sud 1tqj 1vxr 1yjc 2fus 2sgp 3bte 3pro 5sga 1a5h 1arb 1beu 1c1p 1cbx 1d3q 1e5t 1elv 1fph 1gdq 1hax 1im0 1l4z 1myp 1p04 1qbn 1rjx 1sue 1trm 1w4l 1ykf 2g6w 2sgq 3btf 3ptb 5sic 1a5i 1arc 1bfd 1c1q 1ce5 1d3t 1e66 1eoj 1fq3 1gdu 1hay 1inc 1l5h 1mzj 1p05 1qbo 1rp1 1sup 1trn 1w6r 1yme 2gch 2sic 3btg 3ptn 5yas 1a5s 1arl 1bfi 1c1r 1cea 1d4p 1e7u 1eol 1fss 1geq 1haz ldt 1mzs 1p06 1qbv 1rpx 1szb 1try 1w75 1ysc 2gct 2sni 3bth 3qmv 6cha 1a61 1arm 1bfj 1c1s 1ceb 1d5l 1e7v 1ept 1fuj 1gg6 1hb0 1ivy 1lga 1n2c 1p09 1qd5 1rrk 1tab 1ttp 1w76 1yyd 2gft 2st1 3btk 3qmw 6cpa 1a7u 1au8 1bfk 1c1t 1cex 1d6r 1e89 1eq9 1fuo 1ggd 1hbj 1iwb 1lhc 1n6d 1p0i 1qd6 1rs0 1tal 1ttq 1wab 1yyg 2gmt 2sta 3btm 3s8y 6est 1a88 1au9 1bfu 1c1u 1cfj 1d6w 1e8d 1es9 1fup 1gha 1hbt 1iy7 1lhd 1n6e 1p0m 1qe5 1rtf 1taw 1tyf 1wcz 1yyy 2gv6 2stb 3btq 3sc2 6gch 1a8q 1auj 1bhx 1c1v 1cgh 1d7w 1e8m 1esa 1fuq 1ghb 1hdq 1j1i 1lhe 1n6f 1p0p 1qfm 1rtk 1tbr 1tyn 1whs 1yzp 2gv7 2tbs 3btt 3sga 6lpr 1a8s 1auo 1bio 1c1w 1cgi 1d9b 1e8n 1esb 1fur 1gi7 1hdt 1jbv 1lhf 1n8s 1p0q 1qfs 1rw4 1tbs 1u5b 1wht 1yzr 2gyo 2tec 3btw 3sgb 6yas 1a8u 1aur 1bit 1c29 1cgj 1d9i 1e8w 1est 1fuy 1gi8 1hdu 1jbw 1lhg 1nes 1p10 1qgf 1s01 1tbz 1u6e 1wsy 1z5r 2hat 2tga

3cpa 3sgq 7cpa 1a9o 1aut 1bjr 1c2d 1chd 1dcn 1e8x 1eth 1fv9 1gi9 1he8 1jdy 1lka 1nip 1p11 1qgu 1s02 1tec 1u6s 1x7w 1z8g 2hgt 2tgd 3dcn 3sic 7est 1a9p 1auw 1bju 1c2e 1chg 1ddj 1e8y 1etr 1fw2 1gj7 1hee 1jim 1lkb 1nn6 1p12 1qh1 1sbc 1teo 1uag 1x7x 1zen 2hl6 2tgp 3dd5 3so3 7gch 1a9q 1av7 1bjv 1c2f 1cho 1de0 1e8z 1ets 1fw3 1gj8 1hfd 1jjb 1llp 1npm 1p57 1qh8 1sbh 1tfx 1ub7 1x7y 1zg7 2hvv 2tgt 3dea 3t2n 7kme 1a9r 1avg 1bks 1c2g 1clc 1de7 1e90 1ett 1fwy 1gj9 1hgt 1jmy 1lmw 1nrp 1pba 1qhr 1sbi 1tgb 1ubn 1x7z 1zg8 2hvw 2thf 3djy 3tec 7lpr 1a9s 1avt 1bli 1c2h 1cp2 1dfp 1ea5 1eve 1fxj 1gja 1hia 1jqb 1lpa 1nrr 1pbn 1qid 1sbn 1tgc 1ubs 1x80 1zg9 2ix9 2tio 3dkk 3tgi 7yas 1a9t 1avw 1bma 1c2i 1cpa 1dic 1eai 1ex3 1fxu 1gjb 1hja 1jqg 1lpb 1nrs 1pbw 1qie 1sbt 1tgn 1uc4 1xcp 1zjk 2j4c 2tpi 3ef3 3tgj 8cpa 1ab9 1avx 1bml 1c2j 1cpo 1dio 1eas 1exw 1fy4 1gjc 1hlt 1jrs 1lvy 1nsa 1pca 1qif 1sbw 1tgs 1uc5 1xd8 1zlh 2j74 2trm 3esa 3tpi 8est 1abi 1awf 1bmm 1c2k 1cps 1dit 1eat 1ezs 1fy5 1gjd 1hm0 1jrt 1lw6 1nt0 1pcn 1qig 1sca 1tgt 1ucy 1xd9 1zli 2kai 2trs 3esb 3tqx 8gch 1abj 1awh 1bmn 1c2l 1cpx 1dj9 1eau 1ezu 1fy8 1gmc 1hm8 1k32 1lxt 1ntp 1pco 1qih 1scb 1thm 1ula 1xdb 1zow 2lpr 2tsy 3esc 3tvj 8kme 1acb 1ax9 1bmt 1c2m 1cpy 1dje 1eaw 1ezx 1fzz 1gmd 1hm9 1k3u 1m1m 1nzi 1ped 1qii 1scd 1thp 1ulb 1xlu 2abz 2min 2tys 3esd 3uag 8lpr 1acj 1ay6 1boq 1c3l 1cse 1dle 1eax 1f0t 1g1m 1gmh 1hn9 1k62 1m1n 1o3p 1pek 1qij 1scj 1thr 1uma 1xlv 2ace 2nip 2uag 3est 3vgc 9est 1acl 1aye 1bpl 1c3x 1cso 1dlk 1eb8 1f0u 1g20 1gnv 1hnd 1k7e 1m1y 1o5e 1pfx 1qik 1scn 1ths 1uo6 1xlw 2ack 2o8l 2ull 3gbs 3vsb 9lpr 1ad8 1az8 1bra 1c47 1ct0 1dnu 1eb9 1f2s 1g21 1gpk 1hne 1k7f 1m34 1o5f 1pht 1qim 1sel 1tio 1ur0 1xuf 2afh 2ok5 2vgc 3gch 3yas 1ae8 1azw 1brb 1c4g 1ct2 1dnw 1ebl 1f57 1g3b 1gpl 1hnh 1k7w 1m4l 1o5z 1pic 1qix 1sfi 1tj7 1ur4 1xug 2afi 2pk4 2wid 3gct 4cha 1af4 1azz 1brc 1c4u 1ct4 1doj 1ee3 1f5l 1g3c 1gpm 1hnj 1k7y 1max 1o6x 1pjp 1qj1 1sgc 1tju 1urk 1xuh 2afk 2pka 2wif 3hat 4cpa 1afe 1b0e 1bro 1c4v 1cua 1dos 1eea 1f6w 1g3d 1gpn 1hnk 1k8x 1may 1ob0 1pk2 1qj4 1sgd 1tjv 1usw 1xui 2ahb 2pkc 2wig 3hea 4est 1afq 1b0f 1brt 1c4x 1cub 1dpo 1eeh 1f8u 1g3e 1gpz 1hpg 1k8y 1mct 1oce 1pk4 1qj6 1sge 1tjw 1uvo 1xuj 2aip 2pm8 2wij 3hi4 4gch 1agy 1b2i 1bru 1c4y 1cuc 1ds2 1eex 1f92 1g5p 1gqr 1hpj 1k8z 1mcu 1okx 1pkr 1qj7 1sgf 1tld 1uvp 1xuk 2aiq 2pmg 2wik 3hrz 4htc 1aht 1b41 1brw 1c5l 1cud 1dst 1egh 1fc4 1g66 1gqs 1hpk 1k98 1mcv 1ols 1pks 1ql7 1sgn 1tmb 1uvs 1xza 2aj9 2pna 2wil 3hs0 4pnp 1ai8 1b57 1bs0 1c5n 1cue 1dsu 1egm 1fdp 1g95 1gvk 1hpl 1kdu 1mcz 1olu 1pkt 1ql8 1sgp 1tmt 1uvt 1xzb 2alp 2pnb 2win 3i6y 4pro 1aix 1b5g 1bs9 1c5o 1cuf 1dtd 1egq 1ffa 1g97 1gvz 1hrt 1kev 1md7 1olx 1pmk 1ql9 1sgq 1tmu 1uvu 1xzc 2axe 2pni 2wsl 3ia2 4ptp 1ak9 1b7x 1bth 1c5p 1cug 1dtw 1egv 1ffb 1g9i 1gwa 1hut 1kfb 1md8 1oo3 1pml 1qle 1sgr 1tng 1uwc 1xzd 2bce 2prk 2wsy 3il3 4sga 1akn 1b80 1btp 1c5q 1cuh 1dui 1eh5 1ffc 1gba 1gyn 1hv7 1kfc 1mee 1oo4 1pnj 1qmm 1sgt 1tnh 1uza 1xze 2bjh 2pro 2xmb 3il4 4sgb 1aks 1b82 1btu 1c5r 1cui 1dwc 1ei9 1ffd 1gbb 1h1b 1hv9 1kfe 1mhl 1op0 1ppb 1qnj 1sgy 1tni 1v11 1xzf 2btc 2ptc 2xmc 3il5 4tgl 1amh 1b85 1btw 1c5s 1cuj 1dwd 1eja 1ffe 1gbc 1h1l 1hvx 1kfi 1min 1op2 1ppc 1qop 1sib 1tnj 1v16 1xzg 2bza 2ptn 2xmd 3il6 4thn

Table S2 Summary of computational and experimental data of the designed enzymes. The peptide sequences identified by Nano LC-MS/MS were highlighted with an underline. The designed triad and oxyanion hole were in italics and bold.

Description	Scaffold (PDB ID)	Numbers of mutation to scaffold	Protein primary structure	Soluble	Initial activity
1a8uD$_1$	1a8u	30	MGPFITVGQENSTSIDLYYEDHGAGQPVVLIV***HF***AYSGHSWERQSAALLDAGYRVITYDLRGFGQSSQPTTGYDMDTMAADLNTVLETLDLQDAVLVVWM***SG***VGMVARYVSSYGTARIAKVAFLASFEPFLLKTDDNPDGAAPKEFFDGKVAAVKADRYAFYTGMFNLQYNLDENLGTRISEEAVRNSWNTAASGGFFAAAAVLTMMIVDFRADIPRIDVPALILHGTGMV***E***VPIENTARVFHKALPSAEYVEVEGAPLALLWTHAEEVNTALLAFLAK	yes	yes
1jbwD$_1$	1jbw	26	MGNYTETVAYIHSFPRLAKTGDHRRILTLLHALGNPQQQGRYIHVTG***HS***GMILAANAIAHVLEASGLTVGLYTSPFIMRFNERIMIDHEPIPDAALVNAVAFVRAALERLQQQQADFNVTEFEFITALAYWYFRQRQVDVAVIEVGIGGDTDSTNVITPVVSVLIDVTITRQKLLGHTITAIAKHKAGIIKRGIPVVTGNLVPDAAAVVAAKVATTGSQWLRFDRDFSVPKAKLHGWGQRFTYEDQDGRISDLEVPLVGDYQQRNMAIAIQTAKVYAKQTEWPLTPQNIRQGLAASHWPAMLEKISDTPLIVIAWT***MD***EP***A***TNVTITALKQLFSQPITVIAGILAVKMYAAMADRLTAAFSTVYLVPVPGTPRALPEAGYEALHEGRLKDSWQEALAASLNDVPDQPIVITGHLYLASAVRQTLLG	yes	no
1jdyD$_1$	1jdy	21	MVKIVTVKTKAYPDQKPGPYGLRKRVKVFQSSTNYAENFIQSIISTVEPAQRQEATLVVGGAGRFYMKEAIQLIVRIAAANGIGRLVIGQNGILITVAVSCIIRKIKAIGGIILAV***SK***LPGGPNGDFGIKFNISNGGPAPEAITDKIFQISKTIEEYAICPDLKVDLGVLGKQQFDLENKFKPFTVEIVDSVEAYATMLRNIFDFNALKELLSGPNRLKIRIDAMHGAVGPYVKKILCEELGAPANSAVNCVPLEDFGGHHPIPNLTYAADLVETMKSGEHDFGAAFDQ***H***D***E***ANMILGKHGFFVNPSDSVAVIAANIFSIPYFQQTGVRGFARSMPTSGALDRVANATKIALYETPTGWKFFGNLMDASKLSLCGEE***N***FLTGSDHIRAWT	yes	no

			GLWAVLAWLSILATRKQSVEDILKDHWHKFGRNFFTRYDYEEVEAEGATKMMKDLEALMFDRSFVGKQFSANDKVYTVEKADNFEYHDPVDGSVSKNQGLRLIFADGSRIIFRLSGTGSAGATIRLYIDSYEKDNAKINQDPQVMLAPLISIALKVSQLQERTGRTAPTVIT		
1zowD$_1$	1zow	25	MNVGIKGFGAYAPEKIIDNAYFEQFLDTSDEWISKMTGIKERHWADDDQDTSDLAYEASVKAIADAGIQPEDIDMIIVATATGDMPFPTVANMLQERLGTGKVASMDQLAACSGFMYSMITAKQYVQSGDYHNILVVGADKTSKILIMT***E***M***HS***IVMFGDGAGAVIIGEVSEGRGIISYEMGSDGTGGKLWYLDKDTGKV***RM***NAREMWKWWVRIMGDASTRVVEKANLTSDDIDLFIPHQWIIRIMESARERLGISKDKMSVSVNKYGVTSAASIPLSIDQELKNGKLKDDDTIVLVGLTMGLTWGAMTIKWG	yes	no

Table S3 Summary of the peptide sequences identified by NanoLC-MS/MS.

Description	Peptide Mass	Peptide Sequence	Peptide Probability
1a8uD1			
	1163.59	QSAALLDAGYR	94.4%
	878.49	VITYDLR	78.2%
	1002.48	YVSSYGTAR	89.3%
	1793.05	IAKVAFLASFEPFLLK	92.6%
	1480.83	VAFLASFEPFLLK	93.5%
	2562.30	VAFLASFEPFLLKTDDNPDGAAPK	92.6%
	1099.48	TDDNPDGAAPK	76.8%
	1822.80	TDDNPDGAAPKEFFDGK	89.6%
	1209.64	EFFDGKVAAVK	80.1%
	3339.63	VAAVKADRYAFYTGMFNLQYNLDENLGTR	69.1%
	2529.16	YAFYTGMFNLQYNLDENLGTR	94.6%
	802.42	ISEEAVR	85.5%
	2996.61	ADIPRIDVPALILHGTGMVEVPIENTAR	66.4%
	2444.31	IDVPALILHGTGMVEVPIENTAR	94.7%
1jbwD1			
	1914.09	RILTLLHALGNPQQQGR	89.3%
	1757.99	ILTLLHALGNPQQQGR	94.7%
	2290.21	IMIDHEPIPDAALVNAVAFVR	93.4%
	1136.69	LLGHTITAIAK	88.7%
	1401.84	LLGHTITAIAKHK	91.2%
	2017.17	RGIPVVTGNLVPDAAAVVAAK	93.1%
	1861.07	GIPVVTGNLVPDAAAVVAAK	96.0%
	1117.59	VATTGSQWLR	92.2%
	1109.55	FDRDFSVPK	76.8%
	1051.57	AKLHGWGQR	89.0%
	852.43	LHGWGQR	78.3%

	1129.47	FTYEDQDGR	88.7%
	1730.88	ISDLEVPLVGDYQQR	92.8%
	1059.57	NMAIAIQTAK	92.4%
	1481.76	QTEWPLTPQNIR	90.9%
	1537.77	QGLAASHWPAMLEK	86.9%
	2812.49	ISDTPLIVIAWTMDEPATNVTITALK	89.3%
	1797.08	QLFSQPITVIAGILAVK	89.7%
	927.39	MYAAMADR	84.8%
	1888.05	LTAAFSTVYLVPVPGTPR	90.9%
	1511.74	ALPEAGYEALHEGR	95.3%
	3448.76	DSWQEALAASLNDVPDQPIVITGHLYLASAVR	64.3%
1jdyD1			
	1689.88	TKAYPDQKPGPYGLR	80.8%
	1460.74	AYPDQKPGPYGLR	93.6%
	2828.40	VFQSSTNYAENFIQSIISTVEPAQR	78.5%
	1156.62	QEATLVVGGAGR	94.5%
	1509.84	FYMKEAIQLIVR	95.3%
	940.57	EAIQLIVR	77.8%
	841.48	IAAANGIGR	85.3%
	2562.52	KIKAIGGIILAVSKLPGGPNGDFGIK	68.6%
	2434.43	IKAIGGIILAVSKLPGGPNGDFGIK	90.6%
	1040.66	AIGGIILAVSK	94.8%
	2193.25	AIGGIILAVSKLPGGPNGDFGIK	93.6%
	1170.60	LPGGPNGDFGIK	88.5%
	1629.80	FNISNGGPAPEAITDK	97.5%
	2346.22	FNISNGGPAPEAITDKIFQISK	89.4%
	799.48	VDLGVLGK	85.5%
	2315.19	FKPFTVEIVDSVEAYATMLR	90.9%
	2040.02	PFTVEIVDSVEAYATMLR	94.0%
	1080.56	NIFDFNALK	86.7%

	1947.02	NIFDFNALKELLSGPNR	94.3%
	884.47	ELLSGPNR	78.1%
	1625.87	IRIDAMHGAVGPYVK	86.5%
	1356.69	IDAMHGAVGPYVK	95.7%
	2388.04	SGEHDFGAAFDQHDEANMILGK	69.5%
	3280.67	HGFFVNPSDSVAVIAANIFSIPYFQQTGVR	84.1%
	1617.81	SMPTSGALDRVANATK	73.8%
	2877.47	SMPTSGALDRVANATKIALYETPTGWK	67.0%
	1277.66	IALYETPTGWK	91.6%
	1128.53	FFGNLMDASK	92.1%
	2155.23	AWTGLWAVLAWLSILATRK	95.4%
	2027.14	AWTGLWAVLAWLSILATR	95.6%
	1761.91	KQSVEDILKDHWHK	80.5%
	930.50	QSVEDILK	89.6%
	1633.82	QSVEDILKDHWHK	73.7%
	1502.64	YDYEEVEAEGATK	96.7%
	1498.70	MMKDLEALMFDR	93.5%
	1108.52	DLEALMFDR	92.2%
	1527.76	QFSANDKVYTVEK	93.4%
	1778.77	ADNFEYHDPVDGSVSK	94.7%
	877.47	LIFADGSR	78.2%
	1089.58	LSGTGSAGATIR	92.3%
	1457.70	LYIDSYEKDNAK	93.6%
	1029.50	LYIDSYEK	77.2%
	1963.12	INQDPQVMLAPLISIALK	92.1%
	2803.56	INQDPQVMLAPLISIALKVSQLQER	78.6%
	858.46	VSQLQER	78.3%
1zowD_1			
	938.45	GFGAYAPEK	91.3%
	2433.14	IIDNAYFEQFLDTSDEWISK	93.9%

2164.92	HWADDDQDTSDLAYEASVK	95.4%
3813.86	AIADAGIQPEDIDMIIVATATGDMPFPTVANMLQER	62.6%
2004.99	QYVQSGDYHNILVVGADK	95.7%
3031.52	ILIMTEMHSIVMFGDGAGAVIIGEVSEGR	74.3%
1470.67	GIISYEMGSDGTGGK	87.2%
1237.63	LWYLDKDTGK	91.8%
849.40	IMGDASTR	78.3%
836.44	LWYLDK	78.4%
2266.17	ANLTSDDIDLFIPHQWIIR	94.3%
2190.15	YGVTSAASIPLSIDQELKNGK	70.6%
1890.99	YGVTSAASIPLSIDQELK	93.4%
2590.37	LKDDDTIVLVGLTMGLTWGAMTIK	92.6%

Table S4 Enzymatic activity of designed enzyme 1a8uD$_1$–M8 after alanine mutation in residue of catalytic triad.

Mutations	S99A	H31A	E230A
Catalytic activity (U/g)[a]	N/A	N/A	2.81 ± 0.01

[a]Mean ± standard deviation

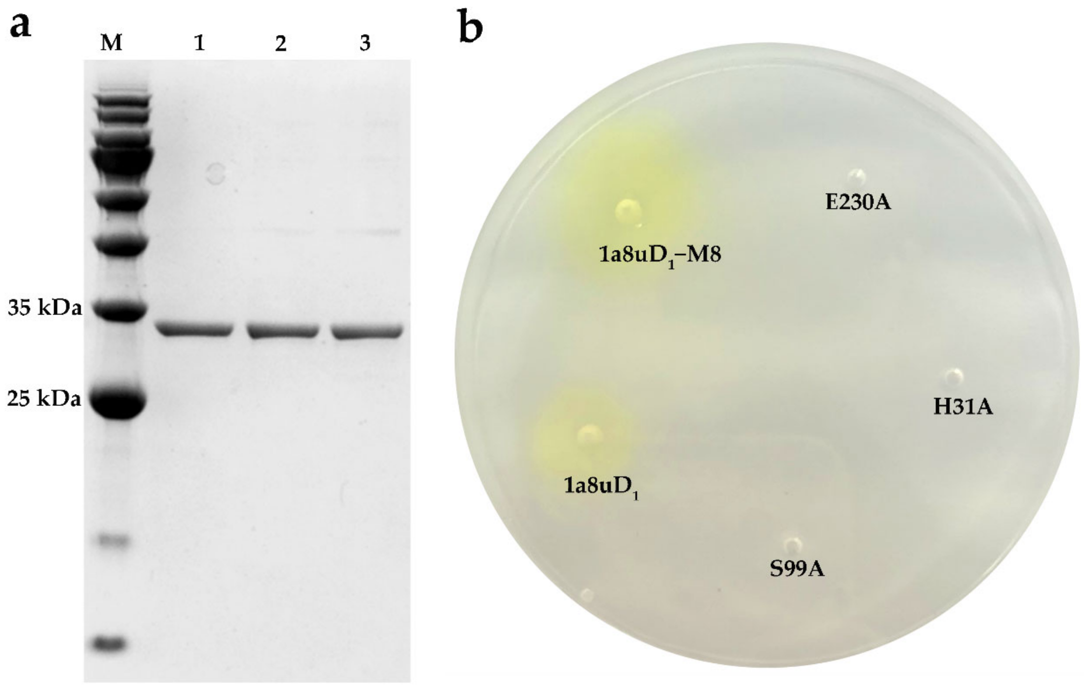

Figure S1 Analysis of purified 1a8uD1–M8 mutants (S99A, H31A, E230A) after alanine mutation of either residue in catalytic triad. (a) SDS-PAGE of purified 1a8uD1–M8 mutants using 15% precast mini polyacrylamide gel. Lane M: standard protein molecular weight (180/140/100/75/60/45/35/25/15/10 kDa), lane 1: mutant S99A, lane 2: mutant H31A, lane 3: mutant E230A. (b) Qualitative analysis of 1a8uD1–M8 mutants according to color change in comparison with that of designed lipases (1a8uD1, 1a8uD1–M8) using p-nitrophenyl octanoate as the substrate.

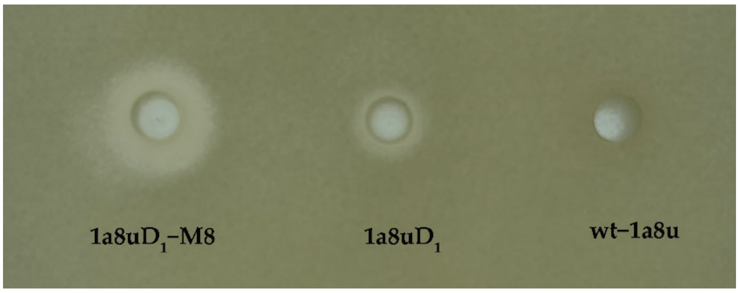

Figure S2 Qualitative analysis of wt–1a8u, mutant 1a8uD1 and mutant 1a8uD1–M8 on a petri dish with emulsified glyceryl trioctanoate (10 mL/L) for 24 h.

Doc S1 Constraint file used for Rosetta Enzyme Design.

```
#block 1 Substrate_Ser_interaction
CST::BEGIN
TEMPLATE:: ATOM_MAP: 1 atom_name: C8 O1 C9
TEMPLATE:: ATOM_MAP: 1 residue3: pNP
TEMPLATE:: ATOM_MAP: 2 atom_type: OH
TEMPLATE:: ATOM_MAP: 2 residue1: S
  CONSTRAINT::   distanceAB:   1.93      0.60    180.   1      5
  CONSTRAINT::   angle_A:      98.07     15.00   100.   360.   3
  CONSTRAINT::   angle_B:      118.90    15.00   100.   360.   3
  CONSTRAINT::   torsion_A:    84.96     20.00   50.    360.   2
  CONSTRAINT::   torsion_AB:   50.32     60.00   50.    360.   2
  CONSTRAINT::   torsion_B:    -152.58   40.00   50.    20.    2
ALGORITHM_INFO:: match
IGNORE_UPSTREAM_PROTON_CHI
ALGORITHM_INFO::END
CST::END

#block 2 Ser_His_interaction
CST::BEGIN
TEMPLATE:: ATOM_MAP: 1 atom_type: OH
TEMPLATE:: ATOM_MAP: 1 residue1: S
TEMPLATE:: ATOM_MAP: 2 atom_type: Nhis
TEMPLATE:: ATOM_MAP: 2 residue1: H
  CONSTRAINT::   distanceAB:   2.53      0.40    100.   0      3
  CONSTRAINT::   angle_A:      116.98    15.00   30.    360.   3
  CONSTRAINT::   angle_B:      133.24    20.00   30.    360.   4
  CONSTRAINT::   torsion_A:    63.70     20.00   50.    360.   2
  CONSTRAINT::   torsion_AB:   73.95     20.00   30.    60.    2
  CONSTRAINT::   torsion_B:    177.28    40.00   30.    360.   2
ALGORITHM_INFO:: match
IGNORE_UPSTREAM_PROTON_CHI
SECONDARY_MATCH: UPSTREAM_CST 1
ALGORITHM_INFO::END
CST::END

#block 3 His_Glu/Asp_interaction
CST::BEGIN
TEMPLATE:: ATOM_MAP: 1 atom_type: Ntrp
TEMPLATE:: ATOM_MAP: 1 residue1: H
TEMPLATE:: ATOM_MAP: 2 atom_type: OOC
```

```
TEMPLATE:: ATOM_MAP: 2 residue1: DE
 CONSTRAINT::    distanceAB:   2.58      0.40    100.    0       2
 CONSTRAINT::    angle_A:      132.06    15.00   30.     360.    3
 CONSTRAINT::    angle_B:      111.31    60.00   30.     360.    3
 CONSTRAINT::    torsion_A:    -179.69   30.00   30.     360.    2
 CONSTRAINT::    torsion_AB:   -179.78   20.00   30.     90.     2
 CONSTRAINT::    torsion_B:    -179.42   20.00   30.     90.     2
ALGORITHM_INFO:: match
IGNORE_UPSTREAM_PROTON_CHI
SECONDARY_MATCH: UPSTREAM_CST 2
ALGORITHM_INFO::END
CST::END

#block 4 oxyanion_hole_1_bb
CST::BEGIN
TEMPLATE:: ATOM_MAP: 1 atom_name: O2 C8 O1
TEMPLATE:: ATOM_MAP: 1 residue3: pNP
TEMPLATE:: ATOM_MAP: 2 atom_type: Nbb
TEMPLATE:: ATOM_MAP: 2 is_backbone
TEMPLATE:: ATOM_MAP: 2 residue1: ACDEFGHIKLMNQRTVWY
 CONSTRAINT::    distanceAB:   3.00      0.30    20.     0       2
 CONSTRAINT::    angle_A:      133.40    60.00   20.     360.    2
 CONSTRAINT::    angle_B:      79.67     20.00   20.     360.    2
 CONSTRAINT::    torsion_A:    79.50     30.00   20.     360.    3
 CONSTRAINT::    torsion_AB:   -139.09   20.00   20.     360.    2
ALGORITHM_INFO:: match
SECONDARY_MATCH: DOWNSTREAM
ALGORITHM_INFO::END
CST::END

#block 5 oxyanion_hole_2_bb
CST::BEGIN
TEMPLATE:: ATOM_MAP: 1 atom_name: O2 C8 O1
TEMPLATE:: ATOM_MAP: 1 residue3: pNP
TEMPLATE:: ATOM_MAP: 2 atom_type: Nbb
TEMPLATE:: ATOM_MAP: 2 is_backbone
TEMPLATE:: ATOM_MAP: 2 residue1: ACDEFGHIKLMNQRTVWY
 CONSTRAINT::    distanceAB:   3.17      0.30    20.     0       2
 CONSTRAINT::    angle_A:      129.87    10.00   20.     360.    2
 CONSTRAINT::    angle_B:      117.87    20.00   20.     360.    2
 CONSTRAINT::    torsion_A:    176.12    20.00   20.     360.    2
 CONSTRAINT::    torsion_AB:   171.07    20.00   20.     360.    2
```

ALGORITHM_INFO:: match
SECONDARY_MATCH: DOWNSTREAM
ALGORITHM_INFO::END
CST::END

Doc S2 The script for Rosetta Enzyme Design.

```
-out:file:silent_struct_type binary
-nstruct 5
-extra_res_fa /storage/enzdes20210311/Rosetta/C8_cst/pNP.params
-ex1
-ex2
-use_input_sc
-linmem_ig 10
-enzdes
  -cstfile /storage/enzdes20210311/Rosetta/C8_cst/enzdes_design.cst
  -detect_design_interface
  -include_catres_in_interface_detection
  -cut1 6.0
  -cut2 8.0
  -cut3 10.0
  -cut4 12.0
  -cst_min
  -cst_opt
  -chi_min
  -cst_design
  -bb_min
  -design_min_cycles 20
  -lig_packer_weight 1.6
  -favor_native_res 0.8
  -start_from_random_rb_conf
  -compare_native ./native_pdbs/
  -final_repack_without_ligand
-packing:soft_rep_design
```
